# The translation elongation factor eEF2 is a novel tumor-associated antigen overexpressed in various types of cancers

**DOI:** 10.3892/ijo.2014.2318

**Published:** 2014-03-04

**Authors:** YUSUKE OJI, NAOYA TATSUMI, MARI FUKUDA, SHIN-ICHI NAKATSUKA, SAYAKA AOYAGI, ERIKA HIRATA, ISAMU NANCHI, FUMIHIRO FUJIKI, HIROKO NAKAJIMA, YUMIKO YAMAMOTO, SYOHEI SHIBATA, MICHIYO NAKAMURA, KANA HASEGAWA, SAYAKA TAKAGI, IKUYO FUKUDA, TOMOKO HOSHIKAWA, YUI MURAKAMI, MASAHIDE MORI, MASAYOSHI INOUE, TETSUJI NAKA, TAKESHI TOMONAGA, YOSHIFUMI SHIMIZU, MASASHI NAKAGAWA, JUNICHI HASEGAWA, RIICHIRO NEZU, HIDENORI INOHARA, SHUICHI IZUMOTO, NORIO NONOMURA, TOSHIKI YOSHIMINE, MEINOSHIN OKUMURA, EIICHI MORII, HAJIME MAEDA, SUMIYUKI NISHIDA, NAOKI HOSEN, AKIHIRO TSUBOI, YOSHIHIRO OKA, HARUO SUGIYAMA

**Affiliations:** 1 Departments of Cancer Stem Cell Biology, Osaka University Graduate School of Medicine, Osaka;; 2 Functional Diagnostic Science, Osaka University Graduate School of Medicine, Osaka;; 3 Cancer Immunology, Osaka University Graduate School of Medicine, Osaka;; 4 Surgery, Osaka University Graduate School of Medicine, Osaka;; 5 Respiratory Medicine and Allergy, Rheumatic Diseases, Osaka University Graduate School of Medicine, Osaka;; 6 Otolaryngology and Sensory Organ Surgery, Osaka University Graduate School of Medicine, Osaka;; 7 Neurosurgery, Osaka University Graduate School of Medicine, Osaka;; 8 Urology, Osaka University Graduate School of Medicine, Osaka;; 9 Pathology, Osaka University Graduate School of Medicine, Osaka;; 10 Cancer Immunotherapy, Osaka University Graduate School of Medicine, Osaka;; 11 Biomedical Informatics, Osaka University Graduate School of Medicine, Osaka;; 12 Department of Pathology, Kansai Rosai Hospital, Hyogo;; 13 Departments of Thoracic Oncology, Toneyama National Hospital;; 14 General Thoracic Surgery, Toneyama National Hospital;; 15 Laboratory of Proteome Research, National Institute of Biomedical Innovation, Osaka;; 16 Department of Internal Medicine, Takarazuka City Hospital, Hyogo;; 17 Department of Internal Medicine, Nissay Hospital;; 18 Department of Surgery, Osaka Rosai Hospital, Osaka, Japan

**Keywords:** eukaryotic elongation factor 2, tumor associated antigen, cytotoxic T lymphocyte, autoantibody, cancer immunotherapy

## Abstract

Recent studies have shown that cancer immunotherapy could be a promising therapeutic approach for the treatment of cancer. In the present study, to identify novel tumor-associated antigens (TAAs), the proteins expressed in a panel of cancer cells were serologically screened by immunoblot analysis and the eukaryotic elongation factor 2 (eEF2) was identified as an antigen that was recognized by IgG autoantibody in sera from a group of patients with head and neck squamous cell carcinoma (HNSCC) or colon cancer. Enzyme-linked immunosorbent assay showed that serum eEF2 IgG Ab levels were significantly higher in colorectal and gastric cancer patients compared to healthy individuals. Immunohistochemistry experiments showed that the eEF2 protein was overexpressed in the majority of lung, esophageal, pancreatic, breast and prostate cancers, HNSCC, glioblastoma multiforme and non-Hodgkin’s lymphoma (NHL). Knockdown of eEF2 by short hairpin RNA (shRNA) significantly inhibited the growth in four eEF2-expressing cell lines, PC14 lung cancer, PCI6 pancreatic cancer, HT1080 fibrosarcoma and A172 glioblastoma cells, but not in eEF2-undetectable MCF7 cells. Furthermore, eEF2-derived 9-mer peptides, EF786 (eEF2 786–794 aa) and EF292 (eEF2 292–300 aa), elicited cytotoxic T lymphocyte (CTL) responses in peripheral blood mononuclear cells (PBMCs) from an HLA-A^*^24:02- and an HLA-A^*^02:01-positive healthy donor, respectively, in an HLA-A-restricted manner. These results indicated that the *eEF2* gene is overexpressed in the majority of several types of cancers and plays an oncogenic role in cancer cell growth. Moreover, the *eEF2* gene product is immunogenic and a promising target molecule of cancer immunotherapy for several types of cancers.

## Introduction

Cancer immunotherapy consists of therapeutic approaches to elicit effective antitumor immunity through active or passive immunization. Recent studies have shown that cancer immunotherapy have potential to provide anticancer activity as a single agent or in combination with conventional surgery, radiation and chemotherapy as reviewed (1–4). These findings indicate that cancer immunotherapy should be a promising therapeutic option for the cancer treatment.

Strategies of cancer immunotherapy include antitumor monoclonal antibodies, cancer vaccines, adoptive transfer of *ex vivo* activated T and natural killer cells, and administration of antibodies or recombinant proteins that either costimulate immune cells or block immune inhibitory pathways (5). Among these strategies, cancer vaccines are approaches to specifically activate host T cells against tumor antigens. The target antigens of cancer vaccine should be: i) highly immunogenic; ii) expressed in a significant proportion of cancer patients; iii) not expressed (or expressed in limited populations) in normal tissues; and iv) required for cancer cell growth and/or survival. Although large number of tumor-associated antigens (TAAs) have been identified using recently developed new technologies such as SEREX and protein microarrays (6,7), there are limited number of antigens that fit all of these criteria in current cancer vaccines.

High level protein biosynthesis is one of the characteristics of cancer cell metabolism (8). Translation is regulated at the initiation and elongation step and deregulated in cancer through a variety of mechanisms (9). Eukaryotic elongation factor 2 (*eEF2*) is a gene that plays an essential role in the polypeptide chain elongation step. Cells control translation levels at elongation step through regulation of eEF2 activity under multiple biological conditions such as cell cycle progression (10) and genotoxic stress (11,12), or in response to endogenous carbon monoxide that exerts antiproliferative effects (13). Previously, we showed that eEF2 was overexpressed in the majority of gastric and colorectal cancers and promoted progression of G_2_/M of the cell cycle in association with activation of Akt and a G_2_/M regulator, cdc2 proteins, resulting in the enhancement of *in vitro* and *in vivo* cancer cell growth (14). However, the role for eEF2 in the tumori-genesis remains largely unknown and it is undetermined whether eEF2 can be a target molecule of molecule-targeted cancer therapy.

In the present study, we identified eEF2 as an antigen eliciting humoral immune responses in a group of patients with HNSCC or colorectal cancer by immunoblot analysis and showed that eEF2 was overexpressed in the majority of various types of cancers such as lung, esophageal, pancreatic, breast and prostate cancers, HNSCC, glioblastoma multiforme and NHL. Knockdown of eEF2 by shRNA significantly inhibited growth of cancer cells. Furthermore, eEF2-derived 9-mer peptides, EF786 (eEF2 786–794 aa) and EF292 (eEF2 292–300 aa), elicited cytotoxic T lymphocyte (CTL) responses in PBMCs from an HLA-A^*^24:02- and an HLA-A^*^02:01-positive healthy donors, respectively, in an HLA-A-restricted manner.

## Materials and methods

### Cell lines

Lung cancer cell lines PC14 and LU99B, pancreatic cancer cell line PCI6, glioblastoma cell line A172, fibrosarcoma cell line HT1080, gastric cancer cell lines MKN28 and AZ-521, and breast cancer cell line MCF7 were cultured in Dulbecco’s modified essential medium supplemented with 10% fetal bovine serum (FBS). Leukemia cell line K562, colon cancer cell line SW480, parent T2 and T2 cells with forced expression of either HLA-A24:02 (T2-2402) (15) or HLA-A02:01 (T2-0201) (16) were cultured in RPMI-1640 medium supplemented with 10% FBS. Leukemia cell line TF-1 was cultured in RPMI-1640 medium supplemented with 10% FBS containing 2 ng/ml human recombinant GM-CSF (Peprotech, Rocky Hill, NJ, USA).

### Sera samples

Sera were obtained from 79 colorectal and 80 gastric cancer patients, 10 patients with head and neck squamous cell carcinoma (HNSCC) and 40 healthy individuals with informed consent at Osaka University Hospital and Osaka Rosai Hospital and stored at −80°C until use.

### Tissue samples

Tumor tissues were obtained from 31 lung adenocarcinoma, 20 small-cell lung cancer, 15 esophageal squamous cell carcinoma, 21 HNSCC, 28 pancreatic cancer, 8 breast cancer, 16 glioblastoma, 4 prostate cancer and 50 NHL (40 diffuse large B-cell lymphoma and 10 folliclular lymphoma) patients. All samples were obtained with informed consent at Osaka University Hospital, Toneyama National Hospital, NHO Osaka Minami Medical Center, and Higashiosaka City General Hospital.

### Western blot analysis

Proteins were separated by SDS-PAGE and transferred to Immobilon polyvinylidene difluoride membrane. After blocking of non-specific binding, the membranes were incubated with the first antibodies, followed by incubation with the corresponding secondary antibodies conjugated with alkaline phosphatase, and visualized using BCIP/NBT kit (Nacalai Tesque, Kyoto, Japan). Polyclonal anti-EF2 (Santa Cruz Biotechnology, Santa Cruz, CA, USA) and anti-GAPDH (Chemicon International, Temecula, CA, USA) were used as the first antibodies.

### Density gradient isoelectric focusing

Density gradient isoelectric focusing was performed by the method reported previously (17) with minor modifications. In brief, K562 cells (5×10^7^ cells) were lysed in 2 ml of 0.1% Triton X-100/PBS. After centrifugation, the supernatant was collected as cytoplasmic fraction. Proteins of the cytoplasmic fraction were precipitated with acetone and the pellet was solved in 1 ml of dH_2_O containing 4% CHAPS and 7 M urea. Isoelectric focusing was carried out using an LKB column (NA-1720, Nihon-Eido Co., Tokyo, Japan) according to the manufacturer’s instructions. On completion of the run, effluent fractions (3 ml each) were collected and twice dialyzed to 200 volume of de-ionized water for 18 h, and then the proteins were precipitated with acetone and stored at −80°C until use.

### MALDI-TOF mass spectrometry

The bands on the silver stained gels were excised with surgical blazor. After dehydration with acetonitrile, the gel slice was dried with Speed Vac. The dried gels were digested with Trypsin (Promega, Madison, WI, USA) at 37°C for 24 h and the tryptic peptides were analyzed. All peptide mass fingerprinting (PMF) spectra were obtained by Matrix-assisted laser desorption/ionization time-of-flight (MALDI-TOF) mass spectrometry using an ultraflex spectrometer (Bruker Daltonics, Bremen, Germany). PMF data were then searched with MS-FIT software against NCBInr database.

### Immunohistochemistry

Formalin-fixed tissue sections were cut from each paraffin-block. After dewaxing and rehydration, the sections were antigen retrieved using Pascal (Dako Cytometry, Glostrup, Denmark) and reacted with the first antibody at 4°C overnight and then reacted with Dako Envision kit/HRP (Dako Cytometry) at room temperature for 30 min. After treatment with 3% H_2_O_2_ solution to reduce endogenous peroxidase activity, immunoreactive eEF2 protein was visualized using diaminobenzidine (DAB). The sections were then counterstained with hematoxylin. The intensity of stain in tumor cells was scored as positive (increased staining in carcinoma cells compared to that in normal cells) or negative (less or negative staining in carcinoma cells) by a pathologist. eEF2-H118 antibody (Santa Cruz Biotechnology) that recognized 741–858aa of eEF2 protein and Sigma-Aldrich #SAB4500695 antibody that recognized the N terminus of eEF2 protein were used as first antibodies. Non-immune rabbit immunoglobulin (Dako Cytometry) was used as negative control for non-specific staining.

### Sequencing

The *eEF2* gene overexpressed in tumors was RT-PCR amplified and directly sequenced in both directions by the method previously described (14).

### Transient expression of shRNA targeting eEF2

Two different shRNA vectors targeting eEF2 mRNA (shEF-1918 and shEF-2804 targeting 1918–1947 and 2804–2833 nt of eEF2 sequence, respectively) were prepared as described previously (14). shRNA targeting luciferase (shLuc) was used as a control. shRNA vectors were transiently expressed as described previously (14).

### Enzyme-linked immunosorbent assay (ELISA)

ELISA was established to measure serum eEF2 IgG Ab levels by a method previously reported (18) with modifications. ELISA 96-well plates were coated with recombinant GST-tagged eEF2 fragmented protein (Ref Seq NM_001961, 411-858 aa) (2 *μ*g/well). Plates were blocked with TBS containing 0.05% Tween-20 and 1% gelatin. Sera were diluted at 1:100 in TBS containing 0.05% Tween-20 (0.05% TBST) and pre-absorbed by immobilized GST protein at 4°C overnight. Then, 100 *μ*l of the diluted sera was added to each well for overnight incubation at 4°C. After washing, captured eEF2 IgG Ab was detected using ALP-conjugated goat anti-human IgG Ab (Santa Cruz Biotechnology) and BCIP/NBT kit. Then, absorbance at 550 nm was measured using a microplate reader. All sera were examined in duplicate. The titers of eEF2 IgG Ab were calculated by interpolation from the standard line which was constructed for each assay from the results of simultaneous measurements of serial dilutions of rabbit polyclonal eEF2 H-118 Ab using the corresponding second Ab (data not shown). eEF2 Ab titer that produces the absorbance at 550 nm equal to that produced by 1.0 *μ*g/ml of eEF2 H-118 Ab in the ELISA system was defined as 1.0 EF2-reacting-unit (ERU).

### Synthetic peptides

The primary amino acid sequence of human eEF2 was analyzed for consensus motifs for 9-mer peptides capable of binding to HLA-A^*^24:02 or 02:01 using ProPred-I computer algorithm (Table I). Then, the top 4 candidate peptides for HLA-A^*^02:01 and 24:02 each were synthesized at immunological grade (Sigma Genosys, Hokkaido, Japan). Synthesized peptide was solved in dH_2_O (2 mg/ml) and stored at −20°C until use.

### MHC stabilization assay

Binding of the synthetic peptides to HLA-A^*^24:02 or 02:01 molecules was evaluated by MHC stabilization assay using antigen processing mutant T2-2402 or T2-0201 cells as described previously (19). Expression of HLA-A24 or HLA-A02 molecules was measured with a FACSort flow cytometer (BD Biosciences, San Jose, CA, USA) and the mean fluorescence intensity (MFI) was recorded.

### In vitro generation of eEF2 peptide-specific CD8^+^ T cells

PBMCs were obtained from an HLA-A^*^24:02-positive and an HLA-A^*^02:01-positive healthy donors by density gradient centrifugation. CD4^+^CD25^+^ Treg cells were depleted from PBMCs by using CD25 MicroBeads (Miltenyi Biotech, Auburn, CA, USA). For generation of autologous dendritic cells (DCs), CD14^+^ monocytes were isolated from the donor PBMCs using BD IMag CD14 isolation kit (BD Bioscience) and cultured in X-VIVO15 (Bio Whittaker, Walkersville, MD, USA) supplemented with 1% human AB serum (Nabi, Miami, FL, USA) containing IL-4 (1,000 U/ml) and GM-CSF (800 U/ml). After 24 h, IL-1β (10 ng/ml), IL-6 (1,000 U/ml), TNF-α (10 ng/ml), and PGE-2 (1 *μ*g/ml) were added to the culture for DC maturation and the cells were cultured for 48 h. DCs were pulsed with EF2 peptide at the concentration of 10 *μ*g/ml in X-VIVO15 supplemented with 1% human AB serum at 37°C for 2 h, irradiated at 30 Gy, and washed 3 times with RPMI-1640 medium. Then, Treg-depleted PBMCs (2×10^6^ cells) were stimulated by co-culture with the EF2 peptide-pulsed DCs at the DC: PBMC ratio of 1:10 in X-VIVO15 supplemented with 5% human AB serum. After 24 h of co-culture, IL-2 (20 U/ml) was added to the culture. The cultured cells were repeatedly stimulated with the EF2 peptide-pulsed, irradiated autologous PBMCs at 10-day intervals. After several times of re-stimulation, the cultured cells were maintained as the established T cell lines in X-VIVO15 supplemented with 5% human AB serum, IL-7 (10 IU/ml) and IL-15 (10 IU/ml) and used for cytotoxic assays.

### ^51^Cr release cytotoxicity assay

Effector cells were prepared from the established T cell lines using Human CD8 T Lymphocyte Enrichment Set-DM (BD Bioscience). Target cells (listed in Table III) were labeled with 100 *μ*Ci of ^51^Cr (Perkin-Elmer, Waltham, MA, USA) at 37°C for 1.5 h and the target cells (1×10^4^ cells) were added to wells containing varying numbers of effector cells in 96-well plates. After 4 h of incubation at 37°C, 100 *μ*l of supernatants were collected from each well and measured for radioactivity. The percentage of specific lysis was calculated as follows: percentage of specific lysis = (cpm of experimental release - cpm of spontaneous release)×100/(cpm of maximal release - cpm of spontaneous release). Radioactivity of the supernatant of the target cells that were cultured without effector cells and the radioactivity of target cells that were completely lysed by the treatment with 1% Triton X-100 was used for spontaneous and maximal release, respectively. The characteristics of target cells in cytotoxicity assay are listed in Table III.

### Statistics

The statistical significance in a difference between arithmetical means of test groups was assessed by unpaired t-test or Kruskal-Wallis test. After Kruskal-Wallis test, Scheffe’s F-test was used as a post hoc test.

## Results

### Production of IgG autoantibody against eukaryotic elongation factor 2 (eEF2) in cancer patients

**Figure 1.**
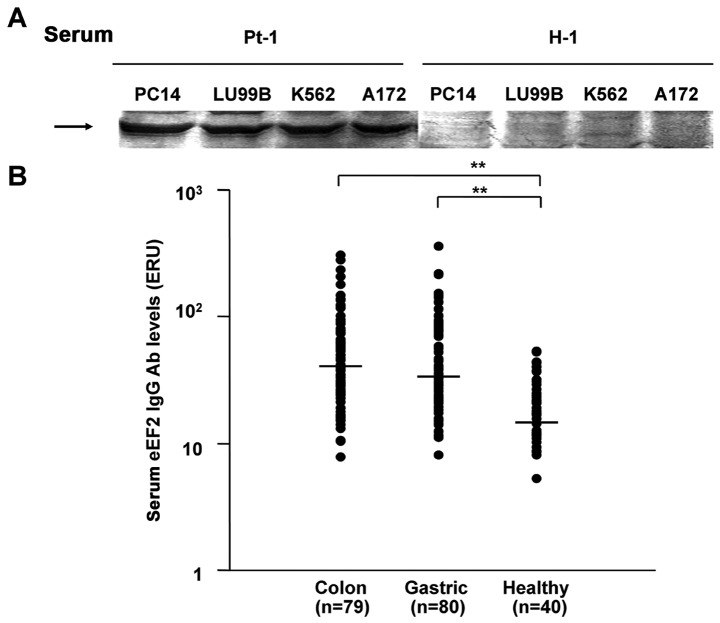
Elevation of serum eEF2 IgG autoantibody levels in cancer patients. (A) Cytoplasmic proteins from PC14, LU99B, K562 and A172 cells were subjected to immunoblot analysis using sera as the first antibodies. Representative results with sera from an HNSCC patient (Pt-1) and a healthy control individual (H-1) are shown. Arrows indicate the protein that is recognized by IgG autoantibody in the sera from the HNSCC patient. (B) Elevation of serum eEF2 IgG autoantibody levels in cancer patients. Assays were performed in duplicate. Colon, colorectal cancer; gastric, gastric cancer; and healthy, healthy individuals. Standard bar represents median value. ^**^p<0.01. eEF2 Ab levels that produces the absorbance at 450 nm equal to that produced by 1 *μ*g/ml of anti-eEF2 H-118 Ab in the ELISA system were defined as 1.0 eEF2-reacting-unit (ERU).

To identify novel tumor-associated antigens (TAAs) with high molecular weight (more than 100 kDa), which were difficult to isolate by standard two dimensional electrophoresis methods because they could not be absorbed into a strip gel, proteins from tumor lysates were first separated by SDS-PAGE, transferred to PVDF membrane, and then probed with sera from tumor-bearing patients. As shown in Fig. 1A, an approximately 100 kDa protein was recognized by sera from 4 of 10 HNSCC and 2 of 3 colon cancer patients in cytoplasmic proteins from two lung cell lines (PC14 and LU99B), one leukemic cell line (K562) and one glioblastoma cell line (A172), whereas it was not recognized by the sera from 5 healthy individuals. To identify this protein, cytoplasmic proteins of K562 cells were fractionated by density gradient isoelectric focusing, separated by SDS-PAGE, and subjected to immunoblot analysis using sera from an HNSCC patient as the first antibody. Since immunoblot analysis detected this protein in fractions of pH 6.62 and pH 6.75, the silver-stained band corresponding to this protein was excised from the SDS-PAGE gel and the protein was analyzed by MALDI-TOF Mass Spectrometry. The search for NCBInr database by MS-Fit software identified the protein as human eukaryotic elongation factor 2 (eEF2) that had M.W. of 95.3-kDa and calculated pI of 6.4.

### Elevation of serum eEF2 IgG antibody levels in cancer patients

Serum eEF2 IgG Ab levels were examined by ELISA in 79 colorectal and 80 gastric cancer patients and 40 healthy individuals and detected in all the samples examined (Fig. 1B). eEF2 IgG Ab levels ranged from 7.8 to 301.7 (median 41.1), from 8.1 to 353.9 (median 33.6) and from 5.2 to 53.0 (median 20.6) ERU in colorectal and gastric cancer patients and healthy individuals, respectively. eEF2 IgG Ab levels were significantly (p<0.01) higher in both colorectal and gastric cancer patients than healthy individuals.

### Overexpression of eEF2 in various types of human cancers

**Figure 2.**
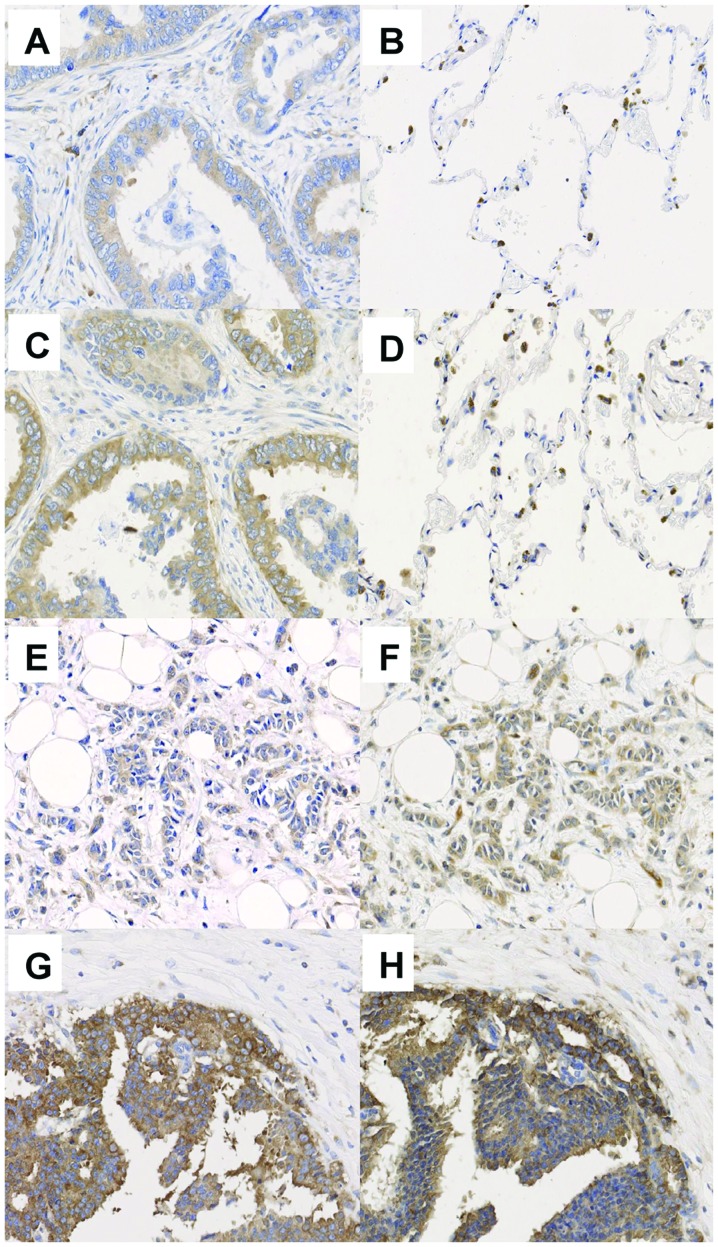
Overexpression of eEF2 in various types of cancers. Representative results of immunohistochemical analysis for eEF2 protein expression in (A and C) lung adenocarcinoma, (B and D) normal lung cells, (E and F) breast cancer, and (G and H) prostate cancer. eEF2 was stained with (A, B, E and G) eEF2-H118 antibody or (C, D, F and H) #SAB4500695 antibody. eEF2 protein was stained brown. Macrophages are non-specifically stained in normal lung tissues.

eEF2 protein was immunohistochemically examined in 51 lung cancers, 15 esophageal squamous cell carcinomas, 21 HNSCCs, 28 pancreatic cancers, 8 breast cancers, 16 glioblastoma multiformes, 4 prostate cancers and 50 NHLs. Immunohistochemical analysis with two different anti-EF2 antibodies recognizing different regions of eEF2 protein showed similar results. Overexpression of eEF2 protein was detected in 71.0% (22 of 31) of lung adenocarcinoma, 95.0% (19 of 20) of small-cell lung cancer, 73.3% (11 of 15) of esophageal cancer, 60.7% (17 of 28) of pancreatic cancer, 50.0% (4 of 8) of breast cancer, 75.0% (3 of 4) of prostate cancer, 52.4% (11 of 21) of HNSCC, 75.0% (12 of 16) of glioblastoma multiformes, and 94.0% (47 of 50) of NHL. Results are summarized in Table I. Representative results are shown in Fig. 2.

### Overexpressed eEF2 gene is a non-mutated, wild-type

To examine whether or not the overexpressed *eEF2* gene was non-mutated, wild-type, the 5′ (84–1334 nt) and the 3′ (1314–2660 nt) sequences of eEF2 mRNA (coding sequence: 84-2660 nt) from five lung adenocarcinomas and five HNSCCs were amplified by RT-PCR and direct sequencing. No mutation was found in the *eEF2* gene in the 10 cancers examined (data not shown).

### Knockdown of eEF2 inhibits cancer cell growth

**Figure 3.**
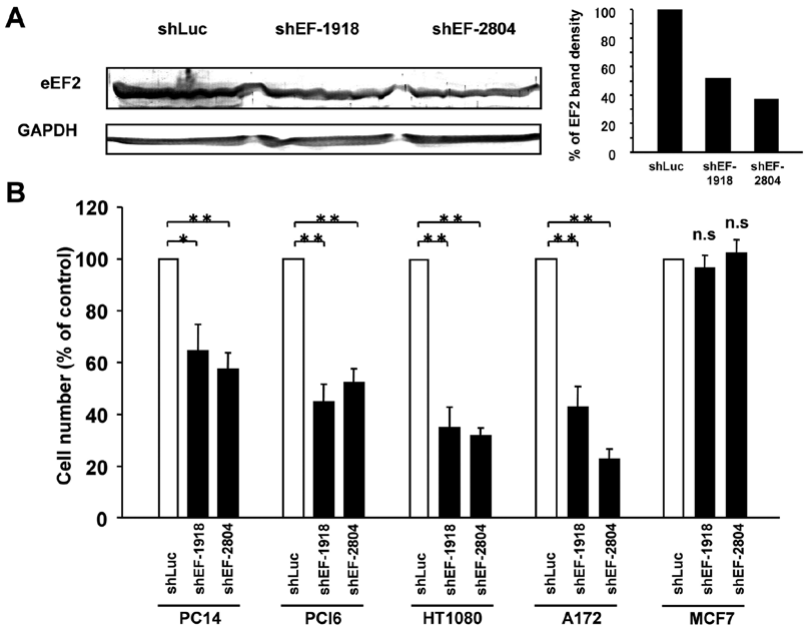
Knockdown of eEF2 inhibits cancer cell growth. Two shRNA vectors targeting different sequences of eEF2 (shEF-1918 and shEF-2804 targeting 1918–1947 and 2804–2833 nt of eEF2 sequence, respectively) or control shRNA targeting luciferase (shLuc) was transfected into PC14, PCI6, HT1080, A172 and MCF7 cells. (A) Reduction in eEF2 protein expression levels in HT1080 cells. Results of western blot analysis are shown. (B) After 72 h of transfection, the cell numbers were examined. ^*^p<0.05; ^**^p<0.01. Experiments were independently performed three times.

To examine the role of eEF2 in cancer cell growth, either of two different shRNAs targeting eEF2 (shEF-1918 and shEF-2804) or a control shRNA targeting luciferase (shLuc) was transfected into four eEF2-expressing cells, lung cancer PC14, pancreatic cancer PCI6, fibrosarcoma HT-1080, and glioblastoma A172 and eEF2-undetectable breast cancer MCF7 cells. After culture for 72 h, both of the two shRNAs targeting eEF2 (shEF-1918 and shEF-2804) reduced eEF2 protein expression levels (Fig. 3A) and significantly inhibited cell growth in all the four eEF2-expressing cells examined (Fig. 3B). However, neither of the two shRNAs targeting eEF2 inhibited growth of eEF2-undetectable MCF7 cells.

### Identification of eEF2 peptides that bind to HLA-A^*^24:02 or HLA-A^*^02:01 molecules

Epitope candidates of eEF2 that bound to HLA-A^*^24:02 or HLA-A^*^02:01 molecules were first analysed using ProPred-I computer algorithm (Table II).

As candidate epitope peptides that bound to HLA-A^*^24:02 molecules, EF78, EF786, EF701 and EF412 peptides were selected and analyzed for binding affinity to HLA-A^*^24:02 molecules by the MHC stabilization assay. These peptides were pulsed to T2-2402 cells and the expression of HLA-A^*^24:02 molecules on the cell surface was analyzed by flow cytometry. As shown in Table II, all the four peptides increased the expression of HLA-A24:02 molecules on T2-2402 cells as a result of the stabilization of HLA-A24:02 molecules. Among the four peptides, EF786 peptide showed binding affinity higher than CMVpp65_328–336_, which was an exogenous cytomegalovirus antigen epitope, to the HLA-A^*^24:02 molecules. As candidate peptides that bound to HLA-A^*^02:01 molecules, EF292, EF739, EF519 and EF671 peptides were selected and analyzed for binding affinity to HLA-A^*^02:01 molecules by the MHC stabilization assay. As shown in Table II, all the four peptides increased the expression of HLA-A02:01 molecules on T2-0201 cells and EF292 peptide showed the highest binding affinity to HLA-A^*^02:01 molecules among the four HLA-A^*^02:01-binding peptides examined.

### Generation of EF2-specific CTLs from HLA-A^*^24:02- or HLA-A^*^02:01-positive donors

Treg-depleted PBMCs from HLA-A^*^24:02- or HLA-A^*^02:01-positive healthy donors were repeatedly stimulated with EF2 peptides (EF786 and EF292 peptides for HLA-A^*^24:02- and HLA-A^*^02:01-positive healthy donors, respectively) and pulsed irradiated autologous DCs and EF2 peptide-specific CTLs were established.

**Figure 4.**
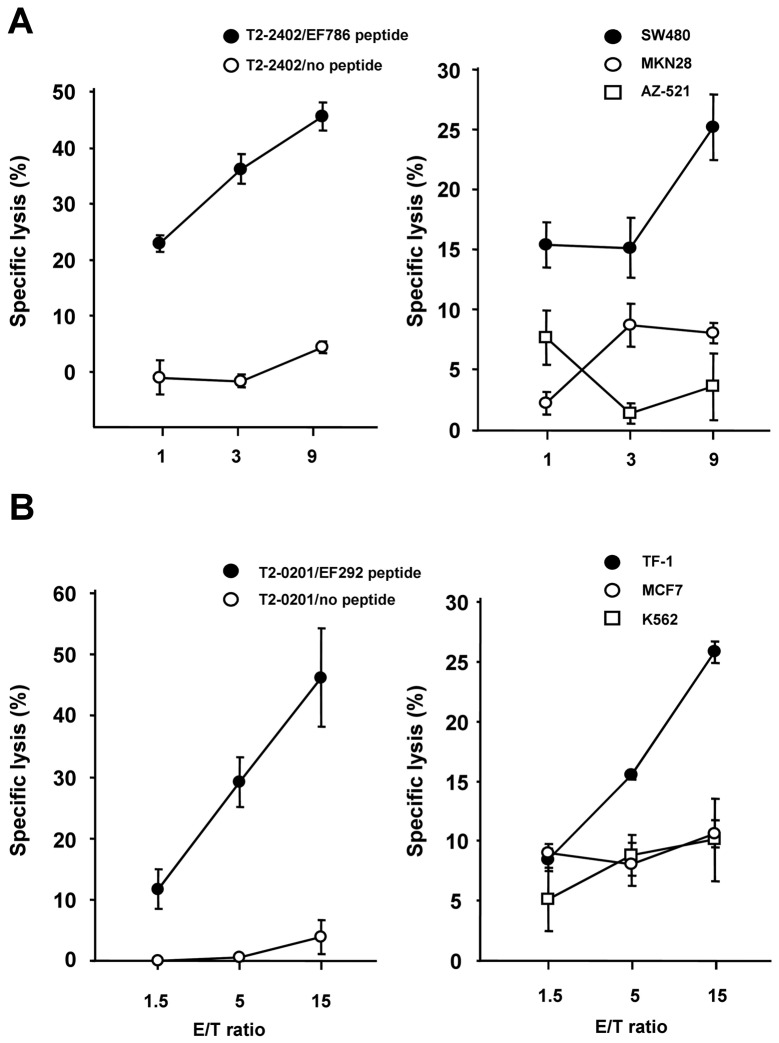
Generation of eEF2-specific CTLs. (A, left panel) Specific lysis of EF786 peptide-pulsed T2-2402 cells by EF786-specific, HLA-A^*^24:02-restricted CTLs. (A, right panel) Specific lysis of eEF2-expressing, HLA-A^*^24:02-positive SW480 by EF786-specific, HLA-A^*^24:02-restricted CTLs. AZ-521 and MKN28 are eEF2-expressing, but HLA-A^*^24:02-negative. (B, left panel) Specific lysis of EF292 peptide-pulsed T2-0201 cells by EF292-specific, HLA-A^*^02:01-restricted CTLs. (B, right panel) Specific lysis of eEF2-expressing, HLA-A^*^02:01-positive TF-1 cells by EF292-specific, HLA-A^*^02:01-restricted CTLs. K562 is eEF2-expressing and HLA-A^*^02:01-negative, and MCF7 is eEF2-undetectable and HLA-A^*^02:01-positive. E/T, effector/target ratio. CTL cytotoxic assays were performed in triplicate.

To examine whether EF2 peptides are capable of eliciting CTL responses, CTL activities of established CTLs were examined. As shown in Fig. 4A, EF786-specific, HLA-A^*^24:02-restricted CTLs lysed EF786 peptide-pulsed T2-2402 cells but not unpulsed ones. The EF786-specific CTLs lysed HLA-A^*^24:02-positive, eEF2-expressing SW480 cells, but not HLA-A^*^24:02-negative, eEF2-expressing AZ-521 and MKN28 cells. As shown in a Fig. 4B, EF292 peptide-specific, HLA-A^*^02:01-restricted CTLs lysed EF292 peptide-pulsed T2-0201 cells but not unpulsed ones. Moreover, the EF292-specific CTLs lysed HLA-A^*^02:01-positive, eEF2-expressing TF-1 cells, but not HLA-A^*^02:01-negative, eEF2-expressing K562 cells and HLA-A^*^02:01-positive, eEF2-undetectable MCF7 cells (Fig. 4B).

## Discussion

We showed that eEF2 was overexpressed in the majority of various types of tumors such as lung, esophageal, pancreatic, and breast cancer and promoted growth of various types of cancer cells. Moreover, *eEF2* gene product elicited both humoral and cellular eEF2-specific immune responses. The production of eEF2 IgG autoantibody was enhanced in patients with colorectal and gastric cancer and 9-mer eEF2 peptides elicited EF2-specific CTLs from healthy donors. These results indicated that overexpressed eEF2 played an oncogenic role and served as a TAA in these tumors.

It is considered that production of autoantibody indicates the potential of its antigen as a target of cancer immunotherapy (20). In the present study, we showed the elevation of serum EF2 IgG levels in colorectal and gastric cancer patients, indicating that eEF2 overexpressed in cancer cells was recognized by the host immune system and induced eEF2-specific immune responses. Since production of IgG autoantibody needed help from CD4^+^ helper T cells (Th cells) for class switch from IgM to IgG, elevation of EF2 IgG Ab levels indicated the activation of EF2-specific Th cells. It is well established that Th cells play an important role in the immune responses against cancer (21). CD4^+^ Th cells are required for activation and maintenance of CD8^+^ CTLs, but they could also exert cytotoxic function against cancer in the absence of CD8^+^ CTLs recognizing antigenic peptides presented by MHC class II molecules (22,23). These results indicated that EF2 protein was an immunogenic molecule that is capable of eliciting not only humoral but also cellular immune responses. In fact, eEF2-derived EF786 peptide showed the binding affinity higher than CMVpp65328-336, an exogenous viral antigen epitope, and elicited *in vitro* EF786-specific CTLs from PBMCs of HLA-A*24:02-positive healthy donors. Taken together, eEF2 protein is highly immunogenic and a promising target molecule for cancer immunotherapy.

Expression of target molecules in tumor cells is the first requisite for TAA-targeting cancer immunotherapy. Survivin is a member of the family of the inhibitor of apoptosis proteins and functions as a key regulator of mitosis and programmed cell death (24). Survivin is overexpressed in various types of tumors with the frequency of 34.5% in gastric cancers (25), 50–60% in colorectal cancers (25,26), 64% in malignant gliomas (27), 53–72% in lung cancers (28,29), and 70.7% in breast cancers (30). Cancer vaccines to induce an antigen-specific immune responses against survivin-expressing tumor cells have been developed with promising results (31,32). Thus, survivin appears to be a promising TAA. However, survivin-targeted immunotherapy may be applicable to a limited population of patients because of its low expression rates in several tumors. In addition, the frequency of survivin-positive tumor cells may vary in individual tumors (25). Thus, the existence of tumor cells lacking survivin could result in tumor evasion from CTL responses against survivin induced by vaccination. NY-ESO-1 is a member of cancer testis antigens and is expressed in a variety of common cancers. Clinical trials that evaluate therapeutic responses against NY-ESO-1 are underway in various cancers (33). However, NY-ESO-1 protein was expressed in only 20 to 30% of lung (34), bladder and ovarian cancers (35) and melanoma and was undetectable in colon and renal cancers (36). Thus, therapeutic strategy against NY-ESO-1 is applicable to a minor population of cancer patients. Compared to these TAAs, eEF2 is more attractive as a target molecule of cancer immunotherapy because of its high frequency of over expression in various types of cancers. The frequency of eEF2 overexpression exceeded 70% in lung, esophageal, breast and prostate cancers, and 90% in gastric and colorectal cancers and NHL, as shown in the present and previous (14) studies. These results indicated that eEF2-targeted immunotherapy should be a therapeutic strategy that would be applicable to the majority of cancer patients. WT1 is also a promising target molecule of immunotherapy and was ranked as top of TAAs (37). WT1 is overexpressed in the majority of leukemia (38) and various types of tumors such as lung (39), colorectal (40) and pancreatic cancer (41), and glioblastoma multiforme (42). However, WT1 might be less expressed in malignant lymphoma. In diffuse large B-cell lymphoma the most common type of NHL, WT1 protein was detected in only 33% of the cases examined (43). Thus, eEF2-targeted immunotherapy may have a priority for NHL.

One mechanism for escape from immune surveillance is the loss of expression of target molecules in cancer cells (44). Therefore, it is important to know whether or not loss of eEF2 expression affects tumor growth in consideration of the potential of eEF2 as a target molecule for cancer immmunotherapy. As shown in the present study, knockdown of eEF2 by shRNA significantly inhibited cancer cell growth. Also, we have demonstrated that eEF2 was overexpressed in the majority of gastric and colorectal cancers and promoted progression of G_2_/M in the cell cycle, resulting in the enhancement of *in vitro* and *in vivo* cancer cell growth (14). Based on these findings showing the involvement of eEF2 in cancer cell growth, it is unlikely that antigenic loss of eEF2 could become a mechanism of tumor escape from eEF2-specific immune responses.

A primary goal of cancer immunotherapy is generation of effective CTL responses through the expansion of robust pre-existing, naturally occurring CD8^+^ CTL precursors and the establishment of long-lasting memory CD8^+^ T cells. This critically depends on the activation of pre-existing antigen-specific CTL precursors as the initial step to induce immune responses. In the present study, eEF2-specific CTL clones were established from HLA-A^*^24:02- or HLA-A^*^02:01-positive healthy donors. In addition, eEF2 IgG autoantibody is detected at low levels in healthy individuals examined. Since these results indicated the existence of not only eEF2-specific CTL precursors but also eEF2-specific B and Th cells even in healthy donors without cancer, the host immune system of cancer patients should have a potential to make robust immune responses against eEF2-expressing cancers by vaccination with EF2 protein or peptide.

In conclusion, eEF2 that is overexpressed in a wide variety of cancers is a promising cancer antigen that can elicit both humoral and cellular immune responses and shows promise as a target molecule of cancer immunotherapy.

## Figures and Tables

**Table I.** Overexpression of eEF2 in human cancers.

Cancer	Overexpression of eEF2 (%)
Lung cancer	80.4 (41/51)
Lung adenocarcinoma	71.0 (22/31)
Small cell lung cancer	95.0 (19/20)
Esophageal squamous cell carcinoma	73.3 (11/15)
Head and neck squamous cell carcinoma	52.4 (11/21)
Pancreatic cancer	60.7 (17/28)
Breast cancer	50.0 (4/8)
Glioblastoma	75.0 (12/16)
Prostate cancer	75.0 (3/4)
Non-Hodgkin’s lymphoma	94.0 (47/50)
Diffuse large B cell lymphoma	92.5 (37/40)
Follicular lymphoma	100 (10/10)

Expression of eEF2 protein in human cancers was examined by immunohistochemistry. Immunostaining was evaluated as positive when cancer cells were stained brown in >10% of the cells.

**Table II.** Characteristics of EF2-derived peptides and results of the MHC stabilization assay.

Peptide	Position (aa)	Sequence	Score	%MFI increase
HLA-A^*^24:02-binding peptides				
EF78	78–86	FYELSENDL	360	40.5
EF786	786–794	AYLPVNESF	252	1552.1
EF701	701–709	RFDVHDVTL	40	297.3
EF412	412–420	AFGRVFSGL	33.6	47.9
CMVpp65 328–336		QYDPVAALF		1344.1
HLA-A^*^02:01-binding peptides				
EF292	292–300	LILDPIFKV	3290	183.3
EF739	739–747	RLMEPIYLV	2426	141.1
EF519	519–527	KLVEGLKRL	705	58.9
EF671	671–679	YLNEIKDSV	642	89.6

The primary amino acid sequences of human eEF2 were analyzed for consensus motifs for 9-mer peptides capable of binding to HLA-A^*^24:02 or 02:01 molecules using ProPred-I software. Percentage MFI increase in MHC stabilization assay was calculated as follows: percentage MFI increase = (MFI with the given peptide - MFI without peptide)/(MFI without peptide) × 100.

**Table III.** Characteristics of target cells in the killing assay.

Target cells	HLA-A^*^24:02 expression	HLA-A^*^02:01 expression	eEF2 expression
T2	−	−	Undetectable
T2-2402	+	−	Undetectable
T2-0201	−	+	Undetectable
SW480	+		+
AZ-521	−		+
MKN28	−		+
TF-1		+	+
K562	−	−	+
MCF7		+	Undetectable

Cell surface protein expression of HLA-A molecules was confirmed by flow cytometry. Expression of eEF2 protein was analyzed by western blot analysis.
